# Self S-RNase reduces the expression of two pollen-specific *COBRA* genes to inhibit pollen tube growth in pear

**DOI:** 10.1186/s43897-023-00074-z

**Published:** 2023-12-01

**Authors:** Lei Wu, Ying Xu, Kaijie Qi, Xueting Jiang, Min He, Yanbo Cui, Jianping Bao, Chao Gu, Shaoling Zhang

**Affiliations:** 1 Centre of Pear Engineering Technology Research, State Key Laboratory of Crop Genetics and Germplasm Enhancement, Nanjing Agricultural University, Nanjing, Jiangsu China; 2 College of Life Sciences, Nanjing Agricultural University, Nanjing, Jiangsu China; 3 Nanjing Ningcui Biological Seed Company Limited, Nanjing, Jiangsu China; 4 College of Plant Science, Tarim University, Alaer, Xinjiang, 843300 China

**Keywords:** *Pyrus*, Self-incompatibility, COBRA, C2H2-type zinc finger protein, Pollen tube growth, S-RNase

## Abstract

Due to self-incompatibility (SI) prevents self-fertilization, natural or artificial cross-pollination has been conducted in many orchards to stabilize fruit yield. However, it is still puzzled which routes of self S-RNase arresting pollen tube growth. Herein, 17 *COBRA* genes were isolated from pear genome. Of these genes, the pollen-specifically expressed *PbCOB.A.1* and *PbCOB.A.2* positively mediates pollen tube growth. The promoters of *PbCOB.A.1* and/or *PbCOB.A.2* were bound and activated by PbABF.E.2 (an ABRE-binding factor) and PbC2H2.K16.2 (a C2H2-type zinc finger protein). Notably, the expressions of *PbCOB.A.1*, *PbCOB.A.2*, and *PbC2H2.K16.2* were repressed by self S-RNase, suggesting that self S-RNase reduces the expression of *PbCOB.A.1* and *PbCOB.A.2* by decreasing the expression of their upstream factors, such as PbC2H2.K16.2, to arrest pollen tube growth. PbCOB.A.1 or PbCOB.A.2 accelerates the growth of pollen tubes treated by self S-RNase, but can hardly affect level of reactive oxygen species and deploymerization of actin cytoskeleton in pollen tubes and cannot physically interact with any reported proteins involved in SI. These results indicate that PbCOB.A.1 and PbCOB.A.2 may not relieve S-RNase toxicity in incompatible pollen tube. The information provides a new route to elucidate the arresting pollen tube growth during SI reaction.

**Graphical Abstract:**

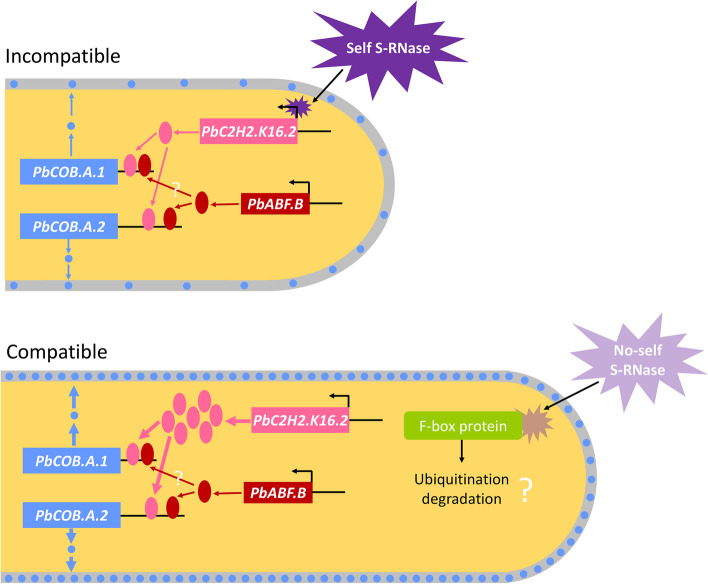

**Supplementary Information:**

The online version contains supplementary material available at 10.1186/s43897-023-00074-z.

## Core

Two *COBRA* genes, *PbCOB.A.1* and *PbCOB.A.2*, positively regulate pollen tube growth in pear. Self S-RNase reduces the expression of *PbCOB.A.1* and *PbCOB.A.2* by decreasing the expression of *PbC2H2.K16.2* (a C2H2-type zinc finger protein) to arrest pollen tube growth.

## Gene & accession numbers

*PbCOB.A.1* genome accession: Pbr004198.1, *PbCOB.A.2* genome accession: Pbr033684.1, *PbABF.E.2* genome accession: Pbr024746.1, *PbC2H2.K16.2*  genome accession: Pbr008219.1. *PbCOB.A.1* NCBI accession: XP_009378852.1, *PbCOB.A.2* NCBI accession: XP_009358118.2, *PbABF.E.2* NCBI accession: XP_048428694.1, *PbC2H2.K16.2* NCBI accession: XP_048439505.1.

## Introduction

Self-incompatibility (SI) is a widespread mechanism that prevents inbreeding and promotes cross-breeding in flowering plants (De Nettancourt et al., 2001). SI systems are classified as sporophytic SI (Anderson et al. 1986; Sassa et al., 1996; Xue et al. 1996) and gametophytic SI (Haasen and Goring 2010; Finnegan et al. 2011). In gametophytic fruit trees, the pistil determinant, a style-specifically expressed S-RNase, can reject self-pollen. The S-RNase is transported into pollen tube by physically interacting with an ATP-binding cassette sub-family F (MdABCF; Meng et al. 2014). Subsequently, self S-RNase depolymerizes the actin cytoskeleton by binding to actin and myosin/villin/GRAM (MVG) proteins (Chen et al. 2018; Yang et al. 2018), and damages vacuole and nuclear by inducing cytoplasmic acidification (Kong et al. 2021). Moreover, self S-RNase can inhibit the activity of a soluble inorganic pyrophosphatase (PPa) by physically interacting with it (Li et al. 2018), and inhibits pollen tube growth by restricting the ABF (ABRE-binding factor)-LRX (Leucine-rich repeat extensin) signaling cascade (Wu et al. 2023), leading to the programmed cell death of self-pollen tube (Wang et al. 2009, 2010).

*COBRA* genes encoding glycosylphosphatidylinositol (GPI)-anchored proteins play the important roles in root and root-hair growth (Hochholdinger et al. 2008; Roudier et al. 2005), biotic and abiotic stresses (Ko et al. 2006; Zaheer et al. 2022), stem strength (Li et al. 2003; Sindhu et al. 2007; Yang et al. 2021), fruit development and ripening (Cao et al. 2012), and pollen tube growth (Li et al. 2013). In *Arabidopsis*, COB1, COB2, COB3, COB4, and COB6 are involved in cellulose deposition in root cells or seed coat cells (Ben-Tov et al. 2015; Brown et al. 2005; Roudier et al. 2002; Schindelman et al. 2001; Sorek et al. 2015). Of these *COB* genes, the first three are necessary for oriented cell expansion in root cells (Schindelman et al. 2001; Roudier et al. 2002). Moreover, COBL5 is associated with pathological resistance (Ko et al. 2006). COBL9 is required for tip-directed growth in root hair development (Parker et al. 2000; Ringli et al. 2005; Jones et al. 2006). COBL10 is reported to be involved in mediating directional growth of pollen tubes, and COBL11 plays a redundant role with COBL10 (Li et al. 2013). However, it is unclear the involvement *COBRA* genes in the S-RNase-based SI reaction.

Zinc finger proteins (ZFPs) contains at least one zinc finger motif that is necessary for DNA binding and protein–protein interaction (Takatsuji 1998). This superfamily can be divided into several families, including C4HC3, C3H, C3HC4, C2HC5, C2H2, C8, C4, C2HC, and C6, based on the number and location of cysteine and/or histidine residues (Berg and Shi 1996). Of these families, C2H2-type ZFPs has been widely studied in flower, leaf, trichome, and fruit developments (Liu et al. 2022). Recently, C2H2-type ZFPs are also reported to be associated with the pollen development and/or pollen tube growth (Arrey-Salas et al. 2021; Lian et al. 2020; Lyu et al. 2019; Puentes-Romero et al. 2022). However, it is unclear the role of C2H2-type ZFPs in the S-RNase-based SI reaction. C2H2-type ZFPs can be involved in plant stress responses (Li et al. 2018; Wang et al. 2019), and bind to the *cis*-acting element GGN(T/g/a/C)V(C/A/g)S(C/G) in the promoter of the target genes to enhance the expression (Feng et al. 2023; Tsutsui et al. 2011). Therefore, a few C2H2-type ZFPs may be responsive to self S-RNase singling to affect pollen tube growth.

Pear crops present the typical gametophytic SI. In the previous studies, we have determined that during SI reaction, self S-RNase binds to actin to deplolymerize the actin cytoskeleton (Chen et al. 2018), and restricts the ABF-LRX signaling cascade to relieve S-RNase cytotoxicity (Wu et al. 2023). In this study, we reported two pollen-specifically expressed *COBRA* genes, *PbCOB.A.1* and *PbCOB.A.2*. They positively stimulated pollen tube growth, and both their promoters were bound and activated by the C2H2-type ZFP, PbC2H2.K16.2. We provide evidence that self S-RNase reduced the expressions of *PbC2H2.K16.2*, *PbCOB.A.1*, and *PbCOB.A.2*. These findings provide a new route to elucidate the arresting pollen tube growth during SI reaction.

## Results

### Identification and expression analysis of *COBRA* genes in pear

**Fig. 1:**
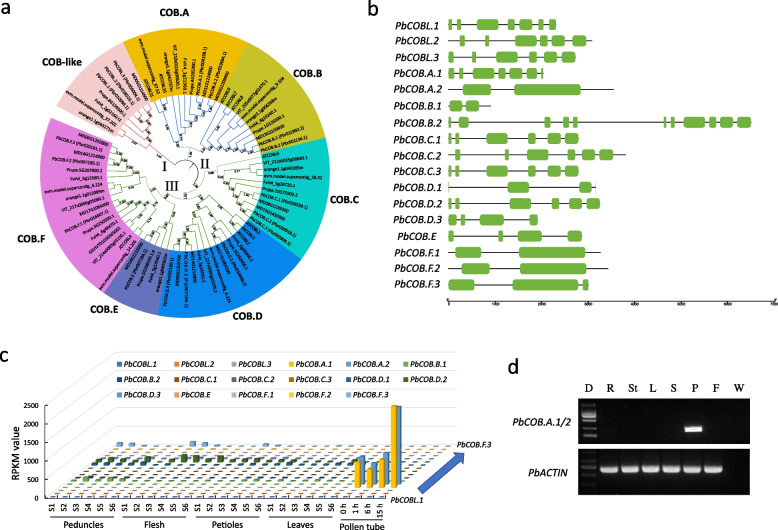
*PbCOB.A.1* and *PbCOB.A.2* are specifically expressed in pollen.** a** Phylogenetic classification of *COBRA* genes in pear, strawberry, peach, apple, papaya, grape, orange, and Arabidopsis. I to III indicate the three classes, respectively. COB.A to COB.F and COB-like are the different groups, respectively. **b** Gene structure of 17 *COBRA* genes in pear genome. Green box represents exon. **c** Transcriptome analysis revealed the expression patterns of 17 *COBRA* genes in pollen tube, peduncles, flesh, petioles, and leaves. S1 to S6 indicate 30, 45, 60, 75, 90, and 110 days after flowering. 0, 1, 6, and 15 h indicate the time pollen cultured in medium. **d** RT-PCR analysis showed the expressions of *PbCOB.A.1* and *PbCOB.A.2* in fruit (F), pollen (P), style (S), leaf (L), stem (St), and root (R). *PbACTIN* acts as a positive control, and water (W) is a negative control. D represents DNA ladder. *PbCOB.A.1/2* indicate both *PbCOB.A.1* and *PbCOB.A.2*

A total of 17 *COBRA* genes were isolated from pear genome (Table S1). Phylogenetic analysis showed that these *COBRA* genes were grouped into three classes (Fig. 1a). Classes II and III were composed by two (COB.A and COB.B) and four (from COB.C to COB.F) groups, respectively (Fig. 1a). Interestingly, the *COBRA* genes in class I contained four exons, the genes in class II contained two or three exons (Fig. 1b). Moreover, most genes in class III contained six exons, while *PbCOB.D.3*, *PbCOB.F.1*, *PbCOB.F.2*, and *PbCOB.F.3* contained eleven, seven, two, and seven exons, respectively (Fig. 1b).

Transcriptome analysis showed that the genes in class I and groups COB.C and COB.E were rarely expressed in all tested tissues (Fig. 1c and Table S1). The genes in groups COB.B, COB.D, and COB.F were expressed in peduncles, flesh, petioles, and leaves, but not in pollen tubes, while the genes (*PbCOB.A.1* and *PbCOB.A.2*) in group COB.A were expressed in pollen tubes, but were almost undetectable in other tissues (Fig. 1c and Table S1). Because the nucleotide sequences of *PbCOB.A.1* had 94.57% identity with those of *PbCOB.A.2* (Fig. S1), and thus a pair of primers were designed to amplify both genes specifically. Semi-quantitative PCR analysis showed that *PbCOB.A.1* and *PbCOB.A.2* were expressed in pollen, but were undetectable in root, stem, leaf, style, and fruit (Fig. 1c). As the control, the *PbACTIN* gene was expressed in all tested tissues (Fig. 1d). These results indicate that PbCOB.A.1 and PbCOB.A.2 may be associated with pollen tube growth.

### Inhibiting of pollen tube growth by knockdown of *PbCOB.A.1* and *PbCOB.A.2*

**Fig. 2:**
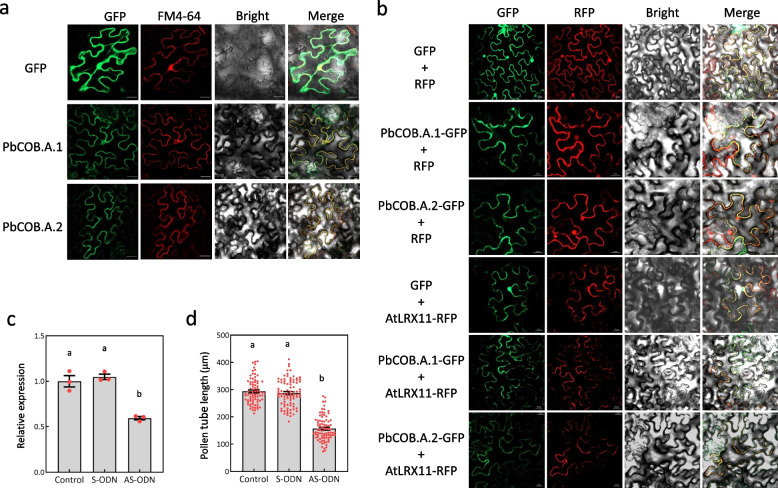
The decreased expression of *PbCOB.A.1* and *PbCOB.A.2* inhibits pollen tube growth.** a** Subcellular localization of PbCOB.A.1- and PbCOB.A.2-GFP fusion proteins. The white line represent a scale (10 μm). FM4-64 is a dye that forces the plasm membrane to present red fluorescence, specifically. **b** The expression level of *PbCOB.A.1* and *PbCOB.A.2* was tested by qRT-PCR in pollen tubes with AS-ODN, S-ODN, or buffer (control) treatment. **c** The length of pollen tubes with AS-ODN, S-ODN, or buffer (control) treatment. Standard errors were calculated from three replicates for qRT-PCR analysis and from at least 90 pollen tubes for pollen tube growth analysis. Analysis of variance was calculated by Student’s *t*-test. Lowercase letters (a and b) indicate *P* < 0.05

To clarify the subcellular localization of PbCOB.A.1 and PbCOB.A.2, the two COBRA proteins and the cell wall-localized AtLRX11 (Fabrice et al., 2018) were individually fused with green fluorescent protein (GFP). The GFP-fused protein was infiltrated into the epidermal cells of tobacco leaves using *Agrobacterium*. As shown in Fig. 2a, because the plasma membrane and endomembrane were dyed by FM4-64 to present red fluorescence, the overlap of red and green fluorescence presented yellow fluorescence. The result showed that green fluorescence was detected on plasma membrane, cell wall, and nucleus in the epidermal cells expressing the GFP protein, and was detected on cell wall, plasma membrane, and endomembrane in the epidermal cells expressing the AtLRX11-GFP, PbCOB.A.1-GFP, or PbCOB.A.2-GFP proteins. Considering the epidermal cells expressing the cell wall-localized AtLRX11-GFP protein also produced the green fluorescence on plasma membrane and endomembrane, PbCOB.A.1 and PbCOB.A.2 are likely localized at cell wall. This result is consistent with the co-localization of AtLRX11-YFP and PbCOB.A.1-GFP or PbCOB.A.2-GFP (Fig. S2).

To test the role of PbCOB.A.1 and PbCOB.A.2 in pollen tube growth, we knocked down the expression of *PbCOB.A.1* and *PbCOB.A.2* using antisense oligodexynucleotide (AS-ODN) experiment. The result showed that at 2 h after treatment (HAT), the expression of *PbCOB.A.1* and *PbCOB.A.2* was decreased in ‘Huanghua’ pollen tubes with the AS-ODN treatment compared to the sense oligodexynucleotide (S-ODN) and buffer (control) treatments (Fig. 2b). Meanwhile, the AS-ODN treatment significantly decreased pollen tube length, compared to the S-ODN or buffer treatment (Fig. 2c). Therefore, the reduced expression of *PbCOB.A.1* and *PbCOB.A.2* inhibits pollen tube growth.

### Accelerating of pollen tube growth by exogenous treatment of PbCOB.A.1 or PbCOB.A.2

**Fig. 3:**
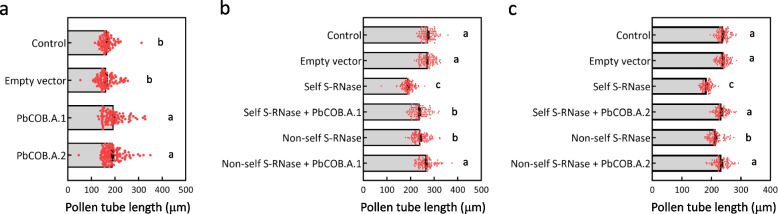
PbCOB.A.1 and PbCOB.A.2 promote pollen tube growth.** a** The length of pollen tubes treated with the recombinant protein of PbCOB.A.1 or PbCOB.A.2, His-tag (Empty vector), and buffer (control). **b** The length of pollen tubes co-treated with the recombinant protein of PbCOB.A.1 and self or non-self S-RNase. **c** The length of pollen tubes co-treated with the recombinant protein of PbCOB.A.2 and self or non-self S-RNase. Standard error was calculated from at least 90 pollen tubes. Analysis of variance was calculated by Student’s *t*-test. Lowercase letters (a and b) indicate *P* < 0.05

To confirm the role of PbCOB.A.1 and PbCOB.A.2 in pollen tube growth, the signal peptide was removed from both the proteins prokaryotic-expressed in *Escherichia coli* (Fig. S3). Using the recombinant proteins to treat pollen tubes of pear cultivar ‘Huanghua’, we found that the exogenous treatment of the recombinant protein of PbCOB.A.1 or PbCOB.A.2 significantly accelerated pollen tube growth, compared to the buffer (control) or His-tag protein (empty vector; Fig. 3a). Therefore, PbCOB.A.1 and PbCOB.A.2 positively accelerate pollen tube growth in pear.

To test the function of PbCOB.A.1 and PbCOB.A.2 against S-RNase toxicity, S-RNase were extracted from the styles of ‘Huanghua’ (*S*_*1*_*S*_*2*_) and ‘Dangshansuli’ (*S*_*7*_*S*_*17*_). The self S-RNase treatment significantly decreased pollen tube length of ‘Huanghua’, compared to the non-self S-RNase and buffer (control) treatments (Fig. 3b, c), suggesting that self S-RNase inhibits pollen tube growth seriously. Notably, when the recombinant protein of PbCOB.A.1 or PbCOB.A.2 was added into the medium together with S-RNase, the pollen tube length were increased, compared to the single S-RNase treatment (Fig. 3b, c). These results indicate that PbCOB.A.1 and PbCOB.A.2 promote the growth of pollen tube treated by self S-RNase.

Self S-RNase can change actin cytoskeleton and ROS (Chen et al. 2018; Wang et al. 2009, 2010). To clarify whether PbCOB.A.1 and PbCOB.A.2 attenuate S-RNase toxicity, we investigated actin cytoskeleton and ROS in pollen tubes with above treatments. The results showed that actin cytoskeleton and ROS concentration were hardly changed in pollen tubes with different treatments, when the recombinant protein of PbCOB.A.1 or PbCOB.A.2 was added into the medium (Fig. S4). The results indicate that PbCOB.A.1 and PbCOB.A.2 cannot attenuate S-RNase toxicity. Taken together, the exogenous treatment of PbCOB.A.1 or PbCOB.A.2 accelerates pollen tube growth but cannot against self S-RNase.

### PbABF.E.2 directly binds to the *PbCOB.A.2* promoter to enhance the activity

**Fig. 4:**
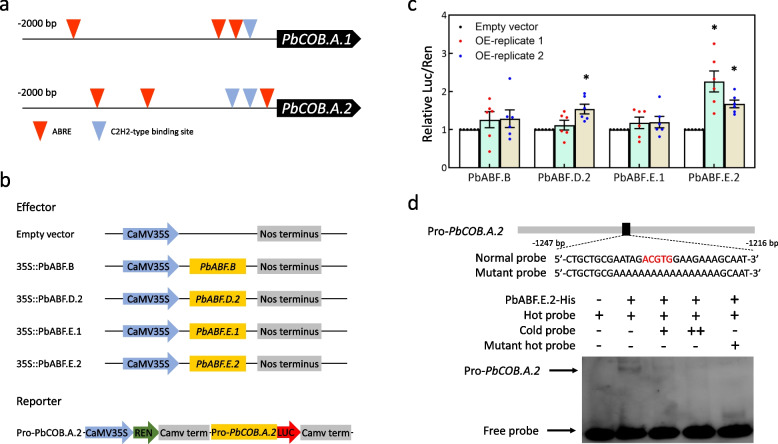
PbABF.E.2 is an upstream factor of *PbCOB.A.2*. **a** The *cis*-elements bound by ABA-binding factor (ABF) and C2H2-type zinc finger protein (ZFP) were predicted from the *PbCOB.A.1* and *PbCOB.A.2* promoters. **b** Reporter and effectors. **c** The *LUC* activities in tobacco leaves over-expressing *PbABF.E.2*, *PbABF.E.1*, *PbABF.D.2*, and *PbABF.B* were evaluated using a dual-luciferase assay. OE-replicate 1 and 2 indicate the two independent experiments. Standard error was calculated from at least five replicates. Analysis of variance was calculated by Student’s *t*-test. Asterisk indicate *P* < 0.05. **d** EMSA assay showing the physical binding of PbABF.E.2 to the *PbCOB.A.2* promoter. ‘–’ and ‘ + ’ indicate the absence and presence of the recombinant PbABF.E.2-His protein, biotin-labeled probe, biotin-labeled mutant, or cold probe, respectively. Cold probe concentrations were tenfold ( +) and 100-fold (+ +) of labeled probes

To identify the upstream factor of *PbCOB.A.1* and *PbCOB.A.2*, we predicted the *cis*-elements from the promoters of both genes and found three ABRE elements (Fig. 4a). It is reported that four *ABF* genes could be detected in pollen grain and pollen tube (Wu et al. 2022, 2023). Each of the four *ABF* genes was inserted into the pSAK277 vector with a CaMV 35S promoter to construct the over-expression vector, while the *PbCOB.A.2* promoter was selected to be inserted into the pGreenII0800-LUC vector (reporter; Fig. 4b). Four effectors and one reporter were used for dual-luciferase assay. The result showed that compared to the empty vector, the *LUC* activity driven by the *PbCOB.A.2* promoter was increased by the over-expression of *PbABF.E.2*, but not changed by the over-expression of *PbABF.E.1*, *PbABF.D.2*, or *PbABF.B* (Fig. 4c). Therefore, only PbABF.E.2 enhances the activity of the *PbCOB.A.2* promoter.

To test the binding of PbABF.E.2 to the *PbCOB.A.2* promoter, the PbABF.E.2 were prokaryotic-expressed in *E. coli* (Fig. S5) and then used for electrophoretic mobility shift assay (EMSA). As a result, the recombinant PbABF.E.2-His protein could bind to the hot probe of the *PbCOB.A.2* promoter (Fig. 4d). In contrast, cold probe could weaken the binding signal, and mutant probe could not be bound by the recombinant PbABF.E.2-His protein (Fig. 4d). This result suggests that PbABF.E.2 directly binds to the *PbCOB.A.2* promoter.

### Reduced expression of *PbCOB.A.1*,* PbCOB.A.2*, and *PbC2H2.K16.2* by self S-RNase

**Fig. 5:**
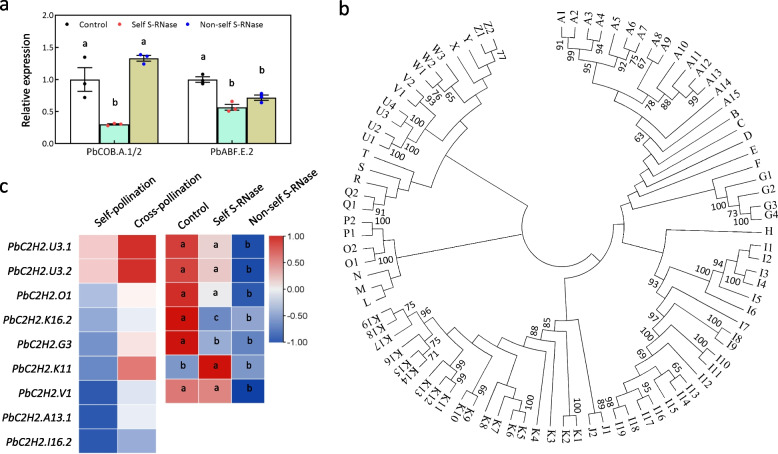
Self S-RNase reduces the expression levels of *PbCOB.A.1*, *PbCOB.A.2*, and *PbC2H2.K16.2*.** a** The expression patterns of *PbABF.E.2*, *PbCOB.A.1*, and *PbCOB.A.2* in the pollen tubes treated with non-self S-RNase, self S-RNase, and buffer (control). **b** Phylogenetic classification of C2H2-type *ZFP* genes in pear, strawberry, papaya, orange, peach, apple, and grape. A1 to A15 are the clusters in group A; B, C, D, E, F, H, L, M, N, R, S, T, X, and Y indicate the corresponding groups; G1 to G4 are the clusters in group G; I1 to I19 are the clusters in the group I; J1 and J2 are the clusters in group J; K1 to K19 are the clusters in group K; O1 and O2 are the clusters in group O; P1 and P2 are the clusters in group P; Q1 and Q2 are the clusters in group Q; U1 to U4 are the clusters in group U; V1 and V2 are the clusters in group V; W1 to W3 are the clusters in group W; Z1 and Z2 are the clusters in group Z. The number above or below the line is the bootstrap value. The accession numbers were listed in Table S2. **c** The left panel showing the differential expression analysis of nine C2H2-type *ZFP* genes in the self-pollinated styles of ‘Dangshansuli’ (self-pollination) and in the ‘Dangshansuli’ styles pollinated with ‘Huanghua’ pollen (cross-pollination). The right panel showing the qRT-PCR analysis of nine C2H2-type *ZFP* genes in the pollen tubes treated with non-self S-RNase, self S-RNase, and buffer (control). Standard error was calculated from three replicates. Analysis of variance was calculated by Student’s *t*-test. Lowercase letters (a and b) indicate *P* < 0.05

To test whether the expression of *PbABF.E.2*, *PbCOB.A.1*, and *PbCOB.A.2* were involved in the SI reaction, self and non-self S-RNase were used to treat the pollen tubes of ‘Huanghua’. The pollen tubes were collected at 0.5 HAT and used for quantitative real-time PCR (qRT-PCR) analysis. As a result, the self S-RNase treatment decreased the expression levels of *PbCOB.A.1* and *PbCOB.A.2*, but could hardly change the expression level of *PbABF.E.2*, compared to the non-self S-RNase and buffer (control) treatments (Fig. 5a). Therefore, only the *PbCOB.A.1* and *PbCOB.A.2* expressions are reduced by self S-RNase.

To identify the bridge factor between self S-RNase and two *COBRA* genes, we further analyzed the *cis*-elements in the *PbCOB.A.1* and *PbCOB.A.2* promoters. The result showed that the *cis*-elements bound by C2H2-type zinc finger proteins (ZFP) were located within both promoters (Fig. 4a). A total of 158 C2H2-type ZFPs were isolated from pear genome. Phylogenetic analysis showed that these *ZFP* genes were grouped into 26 groups, from A to Z (Figs. 5b and S6, Table S2). Based on the transcriptome data in previous report (Shi et al. 2017), we found that 84 *ZFP* genes were expressed (RPKM value > 1) in the self-pollinated styles of ‘Dangshansuli’ (incompatible pollen tubes) and the ‘Dangshansuli’ styles pollinated with ‘Huanghua’ pollen (compatible pollen tubes; Table S3). Further analysis showed that nine *ZFP* genes may be higher expressed in the compatible pollen tubes than in the incompatible pollen tubes (Fig. 5c and Table S3). However, qRT-PCR analysis showed that only *PbC2H2.K16.2* was lower expressed in the pollen tubes with the self S-RNase treatment compared to the non-self S-RNase and buffer (control) treatments (Fig. 5c). Therefore, only the *PbC2H2.K16.2* expression was reduced by self S-RNase.

### PbC2H2.K16.2 directly binds to the *PbCOB.A.1* and *PbCOB.A.2* promoters to enhance the activities

**Fig. 6:**
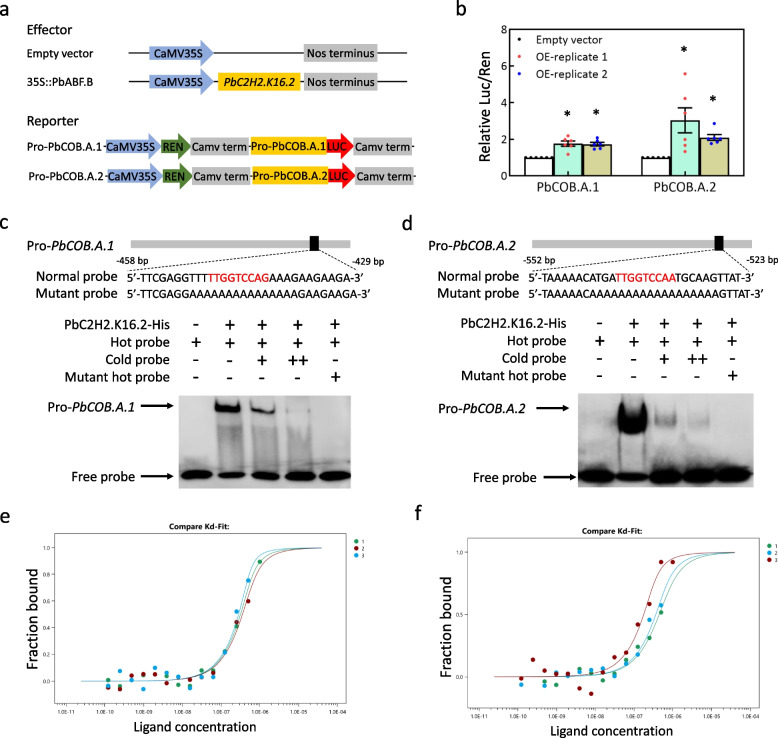
PbC2H2.K16.2 is the upstream factor of *PbCOB.A.1* and *PbCOB.A.2*. **a** Reporters and effector. **b** The *LUC* activities driven by the *PbCOB.A.1* or *PbCOB.A.2* promoter in tobacco leaves over-expressing *PbC2H2.K16.2* were evaluated using a dual-luciferase assay. OE-replicate 1 and 2 indicate the two independent experiments. Standard error was calculated from at least five replicates. Analysis of variance was calculated by Student’s *t*-test. Asterisk indicate *P* < 0.05. EMSA assay showing the physical binding of PbC2H2.K16.2 to the *PbCOB.A.1* (**c**) and *PbCOB.A.2* promoters (**d**). ‘–’ and ‘ + ’indicate the absence and presence of the recombinant PbC2H2.K16.2-His protein, biotin-labeled probe, biotin-labeled mutant, or cold probe, respectively. Cold probe concentrations were tenfold ( +) and 100-fold (+ +) of labeled probes. MST assay showing the binding of PbC2H2.K16.2 to the *PbCOB.A.1* (**e**) and *PbCOB.A.2* promoters (**f**). X-axis represent the concentration gradients of DNA probe, while Y-axis represent the binding capability. The green, cyan, and red color dots represent the three replicates. Each dot represents the binding capacity of PbC2H2.K16.2 to the probe

To test whether PbC2H2.K16.2 enhances the activities of the *PbCOB.A.1* and *PbCOB.A.2* promoters, the full-length sequences of *PbC2H2.K16.2* were inserted into the pSAK277 vector with a CaMV 35S promoter to construct the over-expression vector, while the 2000-bp sequences of the *PbCOB.A.1* and *PbCOB.A.2* promoters were inserted into the pGreenII0800-LUC vector (Fig. 6a). Dual-luciferase assay showed that, compared to the empty vector, the *LUC* activity driven by each promoter was increased by the over-expression of *PbC2H2.K16.2* (Fig. 6b). Therefore, PbC2H2.K16.2 enhances the activities of both promoters.

To test the binding of PbC2H2.K16.2 to the *PbCOB.A.1* and *PbCOB.A.2* promoters, PbC2H2.K16.2 was also prokaryotic-expressed in *E. coli* (Fig. S2). EMSA showed that the recombinant PbC2H2.K16.2-His protein could bind to the hot probes of the *PbCOB.A.1* and *PbCOB.A.2* promoters (Fig. 6c, d). In contrast, cold probe could weaken the binding signal, and mutant probe could not be bound by the recombinant PbC2H2.K16.2-His protein (Fig. 6c, d). Moreover, microscale thermophoresis (MST) assay showed that the binding signal was strengthened with increasing of the DNA probe of the *PbCOB.A.1* or *PbCOB.A.2* promoter (Fig. 6e, f). These results suggest that PbC2H2.K16.2 directly binds to the *PbCOB.A.1* and *PbCOB.A.2* promoters.

## Discussion

### *PbCOB.A.1* and *PbCOB.A.2* are involved in SI by responsive to self S-RNase

During the gametophytic SI reaction, self S-RNase arrests pollen tube growth (Wang et al. 2009, 2010). The pollen tube growth was mediated by many genes, such as *callose synthase 1B.1* (Xia et al. 2023), *rapid alkalinization factor 2* (Kou et al. 2021), and *catharanthus roseus receptor-like kinases 13* (Kou et al. 2022). However, these genes are not associated with the gametophytic SI reaction. Herein, two pollen-specific expressed genes *PbCOB.A.1* and *PbCOB.A.2* were identified from the transcriptome analysis of various tissues in pear (Fig. 1). Both genes are the homologies of *Arabidopsis COBL10* and *COBL11* that are reported to be involved in mediating directional growth of pollen tubes (Li et al. 2013). Coincidently, pollen tube growth was promoted by the exogenous treatment of the recombinant protein of PbCOB.A.1 or PbCOB.A.2 (Fig. 3a), and was inhibited by the knockdown of *PbCOB.A.1* and *PbCOB.A.2* (Fig. 2c). These results suggest that the genes in the group COB.A may positively regulate pollen tube growth. Different from the previous reported genes (Kou et al. 2021, 2022; Xia et al. 2023), the expressions of *PbCOB.A.1* and *PbCOB.A.2* were reduced by self S-RNase compared to non-self S-RNase (Fig. 5a), suggesting that both genes are involved in the SI reaction. The role of both genes is similar to those of the two *LRX* genes reported in a previous study (Wu et al. 2023). In which, both *LRX* genes positively regulated pollen tube growth in pear, but the transcription was reduced by self S-RNase (Wu et al. 2023).

### The potential routes of self S-RNase reducing the *PbCOB.A.1* and *PbCOB.A.2* expression in pear

Self S-RNase arrests pollen tube growth by directly binding to actin to depolymerize actin cytoskeleton (Chen et al. 2018) and inducing cytoplasmic acidification to damage vacuole and nuclear (Kong et al. 2021), leading to the programmed cell death (Wang et al. 2009, 2010). Recently, we found that self S-RNase could reduce the expressions of *PbABF.D.2*, *PbLRXA2.1*, and *PbLRXA2.2* to inhibit pollen tube growth (Wu et al. 2023). Herein, we revealed that self S-RNase could also reduce the *PbCOB.A.1* and *PbCOB.A.2* expression to inhibit pollen tube growth (Fig. 5a). Considering that self S-RNase reduces the *PbLRXA2.1* and *PbLRXA2.2* expression by reducing the *PbABF.D.2* expression (Wu et al. 2023), we speculated that the reduced expression of *PbCOB.A.1* and *PbCOB.A.2* in pollen tube may result from the reduced expression of the upstream factors by self S-RNase. For this reason, we tested the potential interactions between four ABF TFs (Transcription factors) and the *PbCOB.A.2* promoter and found that PbABF.E.2 is the upstream factor of *PbCOB.A.2* (Fig. 4). However, the *PbABF.E.2* expression was hardly mediated by self S-RNase (Fig. 5a). It is reported that exogenous treatment regulates gene expression by mediating the activity of the upstream factors. For example, both CYTOKININ RESPONSE FACTOR4 (MdCRF4) and MCM1-AGAMOUS-DEFICIENS-SRF5 (MdMADS5) are the upstream factors of *1-AMINOCYCLOPROPANE-1-CARBOXYLIC ACID SYNTHASE1* (*MdACS1*) (Li et al. 2023; Xu et al. 2023). Ca^2+^ cannot influence the expression of *MdCRF4* and *MdMADS5*, but can regulate the *MdACS1* expression by promoting CaM2-mediated phosphorylation of MdCRF4 and the CALCIUM-DEPENDENT PROTEIN KINASES7-mediated phosphorylation of MdMADS5 (Li et al. 2023; Xu et al. 2023). Therefore, it is reasonable to speculate that self S-RNase may influence the PbABF.E.2 activity to reduce the *PbCOB.A.2* expression.

Moreover, based on the *cis*-elements predicted from the *PbCOB.A.1* and *PbCOB.A.2* promoters, we also tested the potential interaction between PbC2H2.K16.2 and both promoters, and confirmed that PbC2H2.K16.2 is also the upstream factor of *PbCOB.A.1* and *PbCOB.A.2* (Fig. 6). Notably, the *PbC2H2.K16.2* expression was also reduced by self S-RNase (Fig. 5c). This result indicates that self S-RNase can reduce the *PbCOB.A.1* and *PbCOB.A.2* expression in pollen tube by reducing the expression of their upstream factors, such as *PbC2H2.K16.2*. Taken together, self S-RNase may reduce the *PbCOB.A.1* and *PbCOB.A.2* expression by altering the expression and/or activity of their upstream factors.

### PbCOB.A.1 and PbCOB.A.2 may not attenuate S-RNase toxicity in pollen tube

During the SI reaction, self S-RNase can change the tip-localized ROS gradient and induce the depolymerization of actin cytoskeleton (Chen et al. 2018; Wang et al. 2009, 2010). Herein, we tested the influence of PbCOB.A.1 and PbCOB.A.2 on S-RNase toxicity. However, we found that both PbCOB.A.1 and PbCOB.A.2 could not influence self S-RNase-induced tip-localized ROS disruption and depolymerization of actin cytoskeleton (Fig. S3). Moreover, we surveyed the potential interaction between PbCOB.A.1/PbCOB.A.2 and SI-related factors including self S-RNase (S_1_- and S_2_-RNase), PbABCF, PbMVG, PbActin, PbMYC2, and PbPPa, but no interaction was detected (Fig. S7). These results indicate that PbCOB.A.1 and PbCOB.A.2 regulate pollen tube growth, but could not influence S-RNase toxicity. Therefore, the role of PbCOB.A.1 and PbCOB.A.2 is different from the role of PbLRXA2.1 and PbLRXA2.2 in the SI reaction, because both LRX proteins attenuate S-RNase toxicity by enhancing the stability of actin cytoskeleton (Wu et al. 2023).

In conclusion, we unraveled a new route that self S-RNase reduces the expression of *PbC2H2.K16.2* to decrease the expression of *PbCOB.A.1* and *PbCOB.A.2*, leading to the inhibition of pollen tube growth. In contrast, non-self S-RNase, which may be ubiquitination degraded by pollen-*S* determinant (Sun et al. 2018), cannot influence the expression of *PbC2H2.K16.2*, *PbCOB.A.1*, and *PbCOB.A.2* and pollen tube growth. In future, we will pay more attention to explore how self S-RNase affects the expression of the transcription factors (including PbC2H2.K16.2) involved in pollen tube growth.

## Materials and methods

### Plant materials

Two 8-year-old trees of pear cultivar ‘Huanghua’ (*S*_*1*_*S*_*2*_; *Pyrus pyrifolia*) and three 12-year-old trees of ‘Dangshansuli’ (*S*_*7*_*S*_*17*_; *Pyrus bretschneideri*) were maintained in Baima experimental station of Nanjing Agricultural University (Nanjing, China). The ‘Huanghua’ fruit was collected in August, while the anther, root, stem (young branch), leaf, and style were collected in March. All samples were treated as the described in a previous study (Wu et al. 2023).

### Identification of *COBRA* and C2H2-type *ZFP* genes in pear

Using the *Arabidopsis COBRA* genes as the indexes, blasting analysis was performed in the pear (a woody plant in Maloideae of Rosaceae; http://peargenome.njau.edu.cn/) and other fruit trees. These trees belong to three classifications in Rosid. Apple (*Malus domestica*; a woody plant in Maloideae), peach (*Prunus persica*; woody plant in Prunoideae), and strawberry (*Fragaria* × *ananassa*; an herbaceous plant) are the Rosaceae plants in Fabidae. Papaya (*Carica papaya*) and orange (*Citrus sinensis*) are the Brassicales-Malvales and Citrus plants in Malvidae. Grape (*Vitis vinifera*) belong to an independent classification in Rosid. The genomes of these plants were used online (https://phytozome.jgi.doe.gov/). Moreover, we isolated the C2H2-type *ZFP* genes from pear genome based on the annotation files, and identified the homologies of these genes in the genomes of other fruit trees. Phylogenetic tree was constructed as the described in the previous study (Wu et al. 2023), using the aligned amino acid sequences.

### Subcellular localization

RNA extraction and cDNA synthesis were identical to the previous study (Wu et al. 2023). Using the cDNA of ‘Huanghua’ pollen as the template, the coding sequences of *PbCOB.A.1* and *PbCOB.A.2* were amplified with the primers (Table S4). Moreover, using the cDNA of Arabidopsis pollen as the template, the coding sequences of *AtLRX11* were amplified with the primers (Table S4). The amplified production was inserted into a vector to fuse with the GFP driven by CaMV 35S (Tang et al. 2020). The transference of the constructed vector into leaf and the observation of fluorescence were identical to the previous study (Wu et al. 2023).

### Prokaryotic expression of PbCOB.A.1, PbCOB.A.2, and PbC2H2.K16.2

Both PbCOB.A.1 and PbCOB.A.2 harbor a signal peptide in N-terminal, the sequences without the signal peptide were amplified from the cDNA of ‘Huanghua’ pollen, as well as the coding sequences of *PbC2H2.K16.2* and *PbABF.E.2*. PCR product was ligated with a His-tag protein in the pCold-TF vector to express the recombinant proteins in *E. coli* cells. The expression, extraction and purification conditions were identical to the previously described methods (Chen et al. 2018).

### Pollen culture in vitro

To know down the expression of two *COBRA* genes in pollen tube of Huanghua’, a 24-bp sequences (5’-GTTAAGTTCCAGTGGTCGAGGCGG-3’) were selected for the AS-ODN experiment, and the anti-sense sequences (5’-CCGCCTCGACCACTGGAACTTAAC-3’) were selected for the S-ODN experiment. The treatments are identical to the previously described methods (Chen et al. 2018).

S-RNases were extracted from ‘Huanghua’ and ‘Dangshansuli’ styles, according to the previous studies (Chen et al. 2018; Wang et al. 2009). The treatments and concentrations of S-RNase and recombinant proteins in medium were identical to the previous study (Wu et al. 2023). Reactive oxygen species and actin cytoskeleton in pollen tubes were stained and visualized as the previous study (Wu et al. 2023).

### Protein-DNA interaction

The coding sequences of *PbABF.E.2* and *PbC2H2.K16.2* were amplified from the cDNA of ‘Huanghua’ pollen using the primers (Table S4). PCR product was inserted into the multiple cloning sites of the pSAK277 vector. Meanwhile, Genomic DNA was extracted using DNAsecure Plant Kit (Tiangen, Beijing, China). The 2000-bp sequences of the *PbCOB.A.1* and *PbCOB.A.2* promoters were amplified from ‘Huanghua’ genome with the primers (Table S4). PCR product was inserted into the multiple cloning sites of the pGreenII 0800-LUC vector. Dual-luciferase assay comprised by two independent experiments, and each experiment contained at least five biological replicates. The detail was identical to the previously described methods (Gu et al. 2020).

The binding sites of PbC2H2.K16.2 and PbABF.E.2 were predicted from the promoter sequences of *PbCOB.A.1* and *PbCOB.A.2*. The sequences ranged from -458 to -429 bp of the initiation codon of *PbCOB.A.1* were selected to synthesize the probe biotinylated at 5’ end (Sangon, Shanghai, China). The sequences ranged from -1247 to -1216 bp and from -552 to -523 bp of the initiation codon of *PbCOB.A.2* were individually synthesized into biotinylated probe. These probes were used for EMSA with the recombinant PbC2H2.K16.2 and/or PbABF.E.2. The details of EMSA were identical to the previously described methods (Guo et al. 2021).

Microscale thermophoresis (MST) was performed as the described method in a previous report (Singh et al. 2014). In brief, a total of 500 nM PbC2H2.K16.2-His was labeled using the Monolith NT.115 Protein Labeling Kit RED-NHS (Nanotemper, München, Germany). At least 11 concentration gradients, from 0.001 to 10 μM, were designed for the DNA probes of the *PbCOB.A.1* and *PbCOB.A.2* promoters. Binding reaction and condition were identical to the previous report (Singh et al. 2014). The MST was carried out in the Monolith NT.115 (Nanotemper) and the data was analyzed with Nanotemper analysis software v.1.2.101.

### Protein–protein interaction

The coding sequences of *PbMYC2*, *PbPPa*, *PbMVG*, *PbActin*, *PbABCF*, *PbCOB.A.1*, and *PbCOB.A.2*, were amplified from the cDNA of ‘Huanghua’ pollen with the primers (Table S4), while the coding sequences of *PbS*_*1*_*-* and *PbS*_*2*_*-RNase* were amplified from the cDNA of ‘Huanghua’ style with the primers (Table S4). Each product of *PbCOB.A.1* and *PbCOB.A.2* was inserted into the multiple cloning sites of the pGBKT7 vector (Clontech, PaloAlto, CA), while each product of other genes was inserted into the multiple cloning sites of the pGADT7 vector (Clontech). Y2H was performed as the previous study (Wu et al. 2023).

### Quantitative real-time PCR and statistical analysis

Gene expression level was tested by quantitative real-time PCR (qRT-PCR) with the primers (Table S4). Three replicates were performed. The details were identical to a previous study (Hao et al. 2018). Standard errors were calculated using ANOVA. The significance at *P* < 0.05 was displayed by lowercase letter (such as a and b) or asterisk.

### Supplementary Information

**Additional file 1.**

**Additional file 2.**

## Data Availability

The data supporting the findings of this work are available within the paper and its Supplementary Information.

## Abbreviations

ABCF: ATP-binding cassette sub-family F
ABF: ABRE-binding factor
EMSA: Electrophoretic mobility shift assay
GFP: Green fluorescent protein
GPI: Glycosylphosphatidylinositol
HAT: Hours after treatment
LRX: Leucine-rich repeat extension
LUC: Firefly luciferase
MST: Microscale thermophoresis
MVG: Myosin/villin/GRAM
ODN: Oligodexynucleotide
OE: Over-expression
PPa: Pyrophosphatase
QRT-PCR: Quantitative real-time PCR
REN: Renilla luciferase
RPKM: Reads per kilobase per million
SI: Self-incompatibility
SRK: S-locus receptor kinase
TF: Transcription factor
Y2H: Yeast two-hybrid
ZFP: Zinc finger protein
