# Supplementary material for: Self S-RNase reduces the expression of two pollen-specific COBRA genes to inhibit pollen tube growth in pear
Source: Mol Hortic. 2023 Dec 1;3:26. doi: 10.1186/s43897-023-00074-z (PMC10691131; doi:10.1186/s43897-023-00074-z)
Supplement: Supplementary file 1 — Additional file 1. [file 43897_2023_74_MOESM1_ESM.docx]

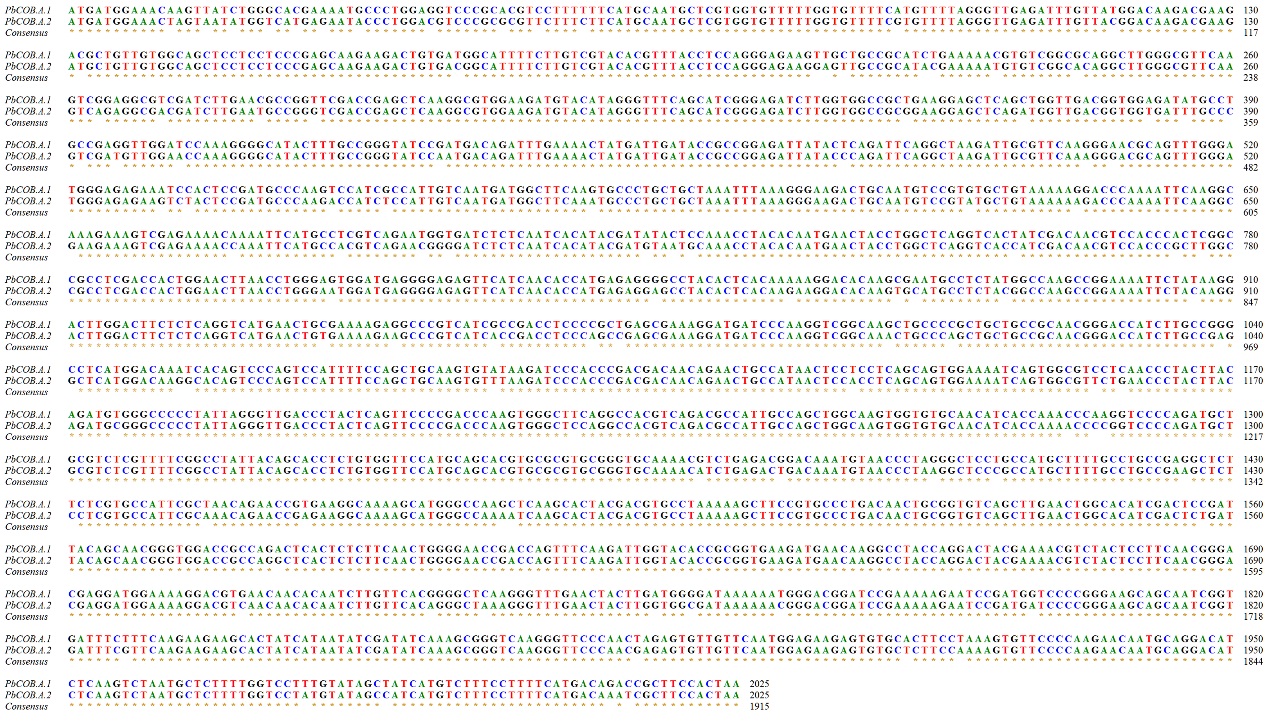


**Figure S1.** Sequence alignment of *PbCOB.A.1* and *PbCOB.A.2*.


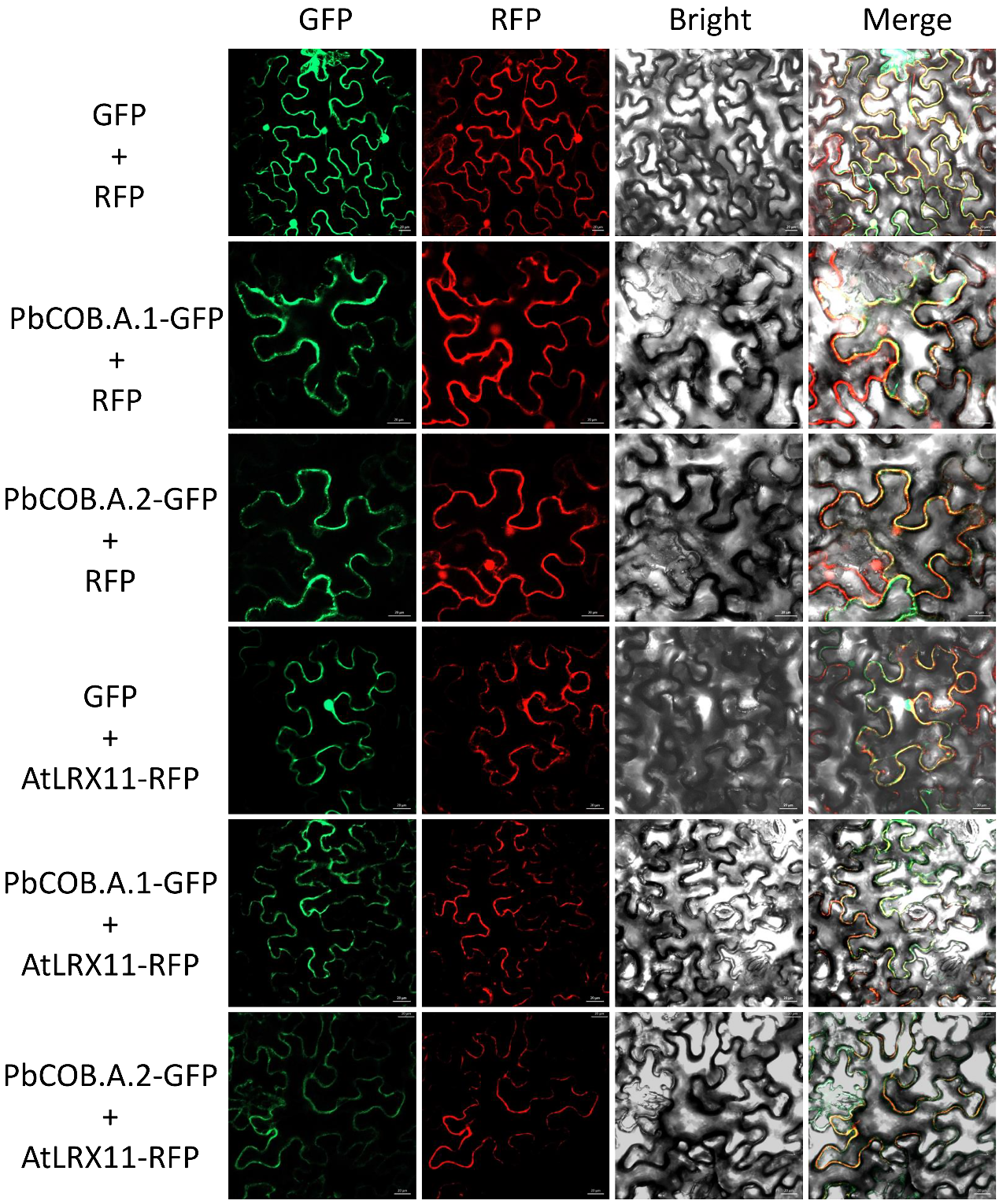


**Figure S2.** Co-localization of AtLRX11-YFP and PbCOB.A.1-GFP or PbCOB.A.2-GFP in tobacco epidermal cells.


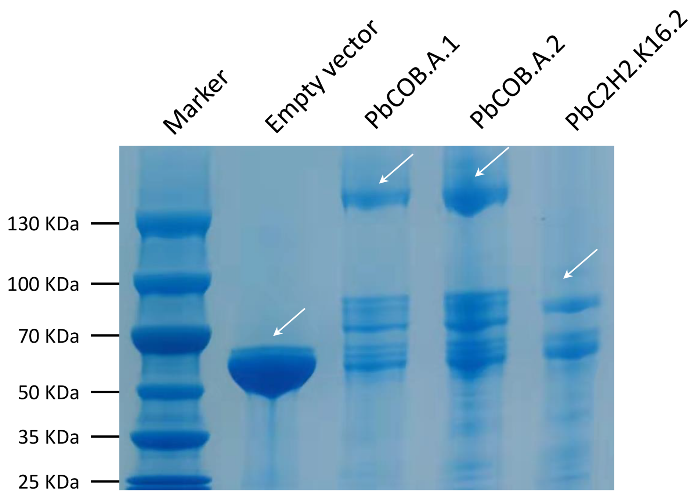


**Figure S3.** Prokaryotic expression of PbCOB.A.1, PbCOB.A.2, and PbC2H2.K16.2 from *Escherichia coli* cells. White arrow represents the target protein.


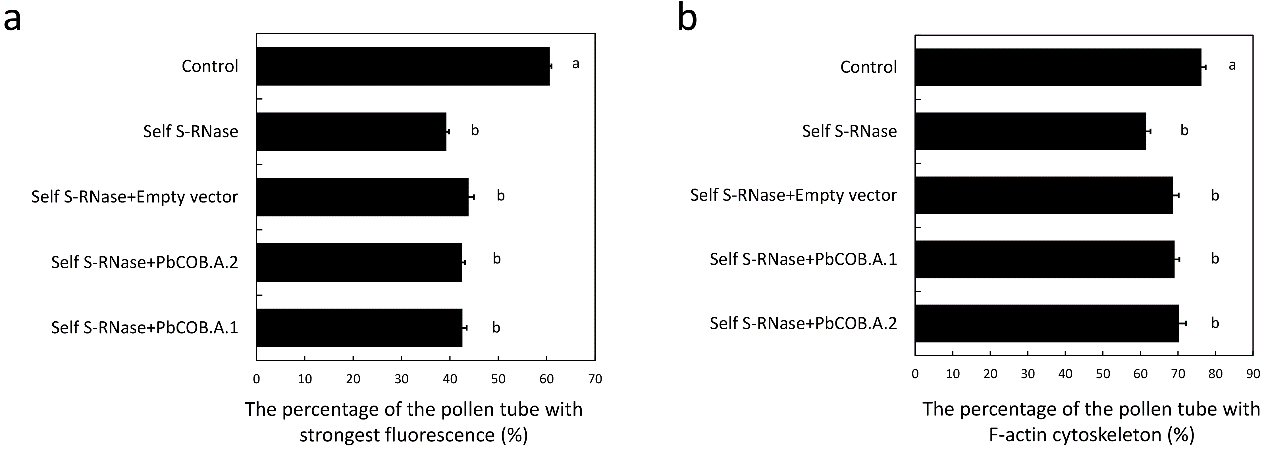


**Figure S4.** PbCOB.A.1 and PbCOB.A.2 cannot affect self S-RNase-induced ROS disruption and deploymerization of actin cytoskeleton. The percentage of the pollen tube with strongest fluorescence (**a**) or with F-actin cytoskeleton (**b**) was calculated from the pollen tubes co-treated with self S-RNase and PbCOB.A.1 or PbCOB.A.2. Standard error was calculated from at least 90 pollen tubes. Analysis of variance was calculated by Student’s t-test. Lowercase letters (a and b) indicate *P* < 0.05.


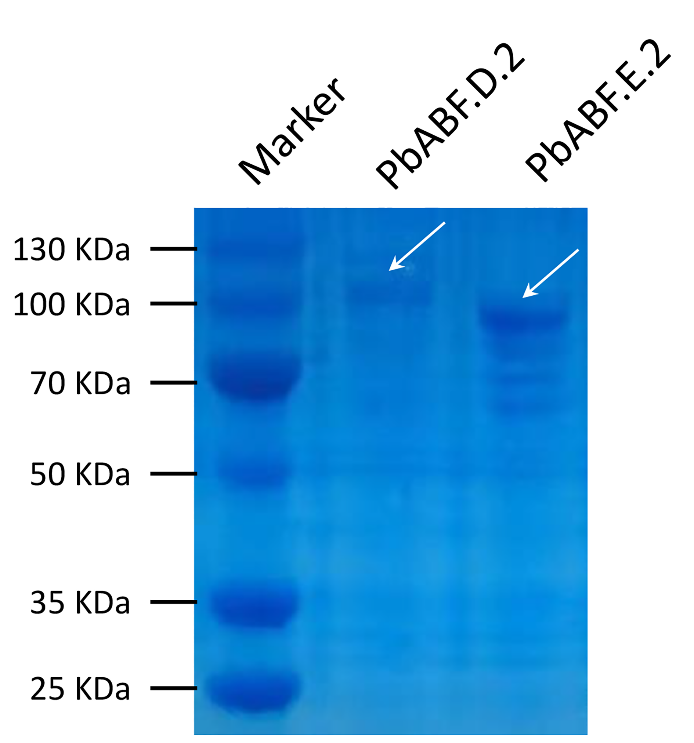


**Figure S5.** Prokaryotic expression of PbABF.E.2 from *Escherichia coli* cells. White arrow represents the target protein.


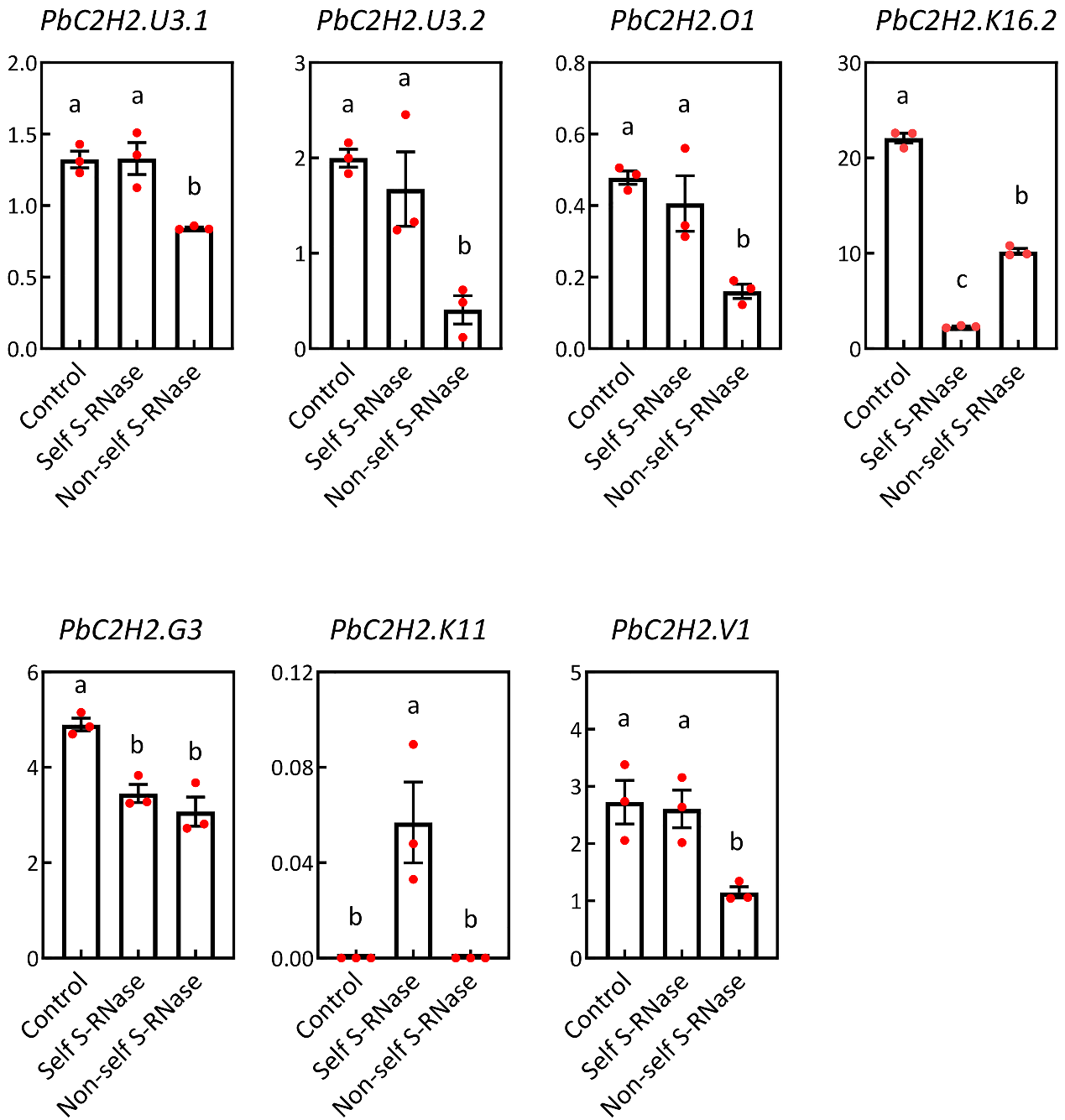


**Figure S6.** The expression levels of C2H2-type *ZFP* genes in the pollen tubes treated by buffer, self S-RNase, and non-self S-RNase. Standard errors were calculated from three replicates. Analysis of variance was calculated by Student’s *t*-test. Lowercase letters (a and b) indicate *P* < 0.05.


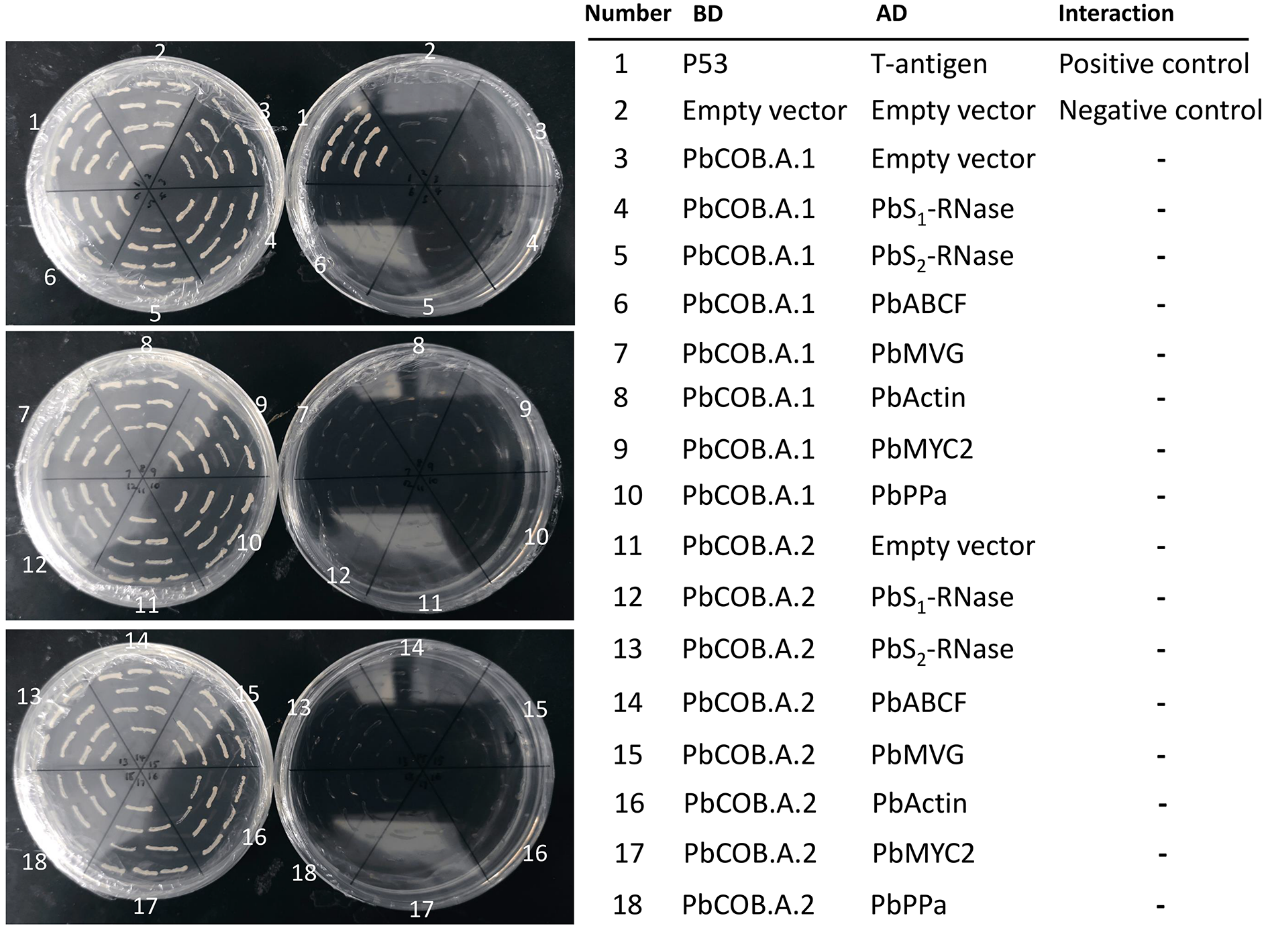


**Figure S7.** PbCOB.A.1 and PbCOB.A.2 could not interact with the reported proteins associated with the GSI reaction. PbABCF, PbMVG, PbPPa, and PbMYC2 are the homologies of MdABCF, MdMVG, MdPPa, and MdMYC2 in apple, respectively. DDO, SD medium lacking Trp and Leu; QDO, SD medium lacking Trp, Leu, His, and Ade. A positive control was designed as the P53 and T-antigen, while a negative control was designed as AD and BD vectors (Empty). ‘-’ represents non-interaction.
